# The effects of substitute multisensory feedback on task performance and the sense of presence in a virtual reality environment

**DOI:** 10.1371/journal.pone.0191846

**Published:** 2018-02-01

**Authors:** Natalia Cooper, Ferdinando Milella, Carlo Pinto, Iain Cant, Mark White, Georg Meyer

**Affiliations:** 1 Construction Research Centre, National Research Council, Ottawa, Canada; 2 UKAEA, Culham Science Centre, Abingdon, United Kingdom; 3 Virtual Engineering Centre, Daresbury, United Kingdom; 4 Department of Psychology, University of Liverpool, Liverpool, United Kingdom; University of Bath, UNITED KINGDOM

## Abstract

Objective and subjective measures of performance in virtual reality environments increase as more sensory cues are delivered and as simulation fidelity increases. Some cues (colour or sound) are easier to present than others (object weight, vestibular cues) so that substitute cues can be used to enhance informational content in a simulation at the expense of simulation fidelity. This study evaluates how substituting cues in one modality by alternative cues in another modality affects subjective and objective performance measures in a highly immersive virtual reality environment. Participants performed a wheel change in a virtual reality (VR) environment. Auditory, haptic and visual cues, signalling critical events in the simulation, were manipulated in a factorial design. Subjective ratings were recorded via questionnaires. The time taken to complete the task was used as an objective performance measure. The results show that participants performed best and felt an increased sense of immersion and involvement, collectively referred to as ‘presence’, when substitute multimodal sensory feedback was provided. Significant main effects of audio and tactile cues on task performance and on participants' subjective ratings were found. A significant negative relationship was found between the objective (overall completion times) and subjective (ratings of presence) performance measures. We conclude that increasing informational content, even if it disrupts fidelity, enhances performance and user’s overall experience. On this basis we advocate the use of substitute cues in VR environments as an efficient method to enhance performance and user experience.

## Introduction

Virtual reality (VR) environments are commonly used for training, research, interpersonal communication, data visualisation and many other purposes [1–4] and as such a continuous optimisation and evaluation of the effectiveness of these systems is required. One of the fundamental decisions designers of VR systems have to make is to select what sensory modalities can be stimulated. Visual and auditory signals are routinely presented, but other signals, in particular vestibular cues, which require complex motion platforms, or haptic cues are often omitted.

There is substantial evidence that improvements in simulation fidelity, the degree to which VR technology delivers an objectively measurable match to real-world sensory signals, (Immersion, in Slater’s terminology [5–7]), improves performance in VR training environments [8–12]. It is therefore tempting to stimulate as many sensory modalities as possible to provide the greatest possible simulation fidelity. Practical considerations, but also financial and operational constraints, limit the degree to which sensory stimuli can be provided in many applications [13]. While there, for example, is progress in providing haptic cues [1–4,14–26] there still are significant challenges [14, 27] that can make some tasks more difficult and decrease overall task efficiency [15] when haptic cues are presented.

Virtual immersive experiences do not just depend on sensory factors, but also include actional and symbolic components to give participants the impression that they are present in the virtual environment [9]. Ferwerda [28] in the context of image fidelity, for example, makes a distinction between physical fidelity (veridical stimulation of the sensory system), photo-realism (veridical representation) and functional fidelity (veridical representation of the symbolic information) and stresses that functional fidelity is particularly task-relevant [11, 28].

In VR environments it is entirely possible to represent functional information in arbitrary signals and modalities. In an assembly task, for example, it may be necessary to fully insert a part before the next step can be undertaken. In real life this information (part snaps into place) would be signalled by a simultaneous change in a range of sensory signals such as weight, vibration, sound or visual cues. Since haptic cues are much more difficult to realise in VR environments than visual or auditory cues [8, 9, 11, 15–20], limiting information to a subset of modalities may not affect performance.

In virtual environments, however, cues are not constrained by the physical limitations of the real world; symbolic information can be presented in arbitrary modalities and as arbitrary signals. The symbolic information of a part ‘snapping into place’, for example, could be represented by a colour change in the part, arbitrary sound or vibration signals. Presenting task-relevant information using alternative signals or modalities means that sensory fidelity reduces because the presented sensory signals no longer objectively match real environments or real behaviour [5–7]. It might therefore be argued that substitute cues should consequently have a negative impact on user experience and performance [8].

An alternative argument is that providing task-relevant information as substitute cues, while reducing physical fidelity, adds functional information that is relevant for natural interaction, which is the focus of Witmer and Singer’s [29–31] definition of immersion: being able to affect, and being affected by, the virtual environment. The provision of task-relevant information in substitute modalities, therefore, may enhance interaction and involvement in virtual environments and consequently may have a positive effect on performance and user experience.

The principal question driving this research is whether cues used in VR environments can be substituted by alternative information-bearing signals or cues in other modalities. While such substitute cues enhance information available to VR users, they negatively impact on the fidelity of the VR environment, which has also been linked to task performance [13, 32–34]. We use subjective measures of user experience and objective measures of task performance to address this question. We hypothesise that substitute sensory cueing will have a positive effect on objective and subjective measures collected from participants during the completion of a realistic task in a VR environment.

In the following sections, we will review the role of multimodal feedback on task performance in VR environments. Then we will discuss previous work on the effects of sensory feedback on user’s sense of presence and immersion, specifically focusing on the relationship between the objective and subjective measures of performance.

### Performance improvements via multi-sensory cues

In interacting with real world environments, we have a bias towards vision as we take most of our information about the environment through the visual modality. In VR environments it is possible to provide additional information in order to improve the ability to identify the important information relevant to the task [35]. Experimental research in multisensory stimulation suggests the potential benefits of other modalities, such as audio and tactile feedback [11, 14, 35–41]. Indeed, studies have shown that visual and audio sensory cues, presented alone or in a combination, improve task performance in target localisation [35], target accuracy [14] and spatial attention tasks without affecting perceptual workload [36–40]. Behavioural data from Meyer et al. [39] revealed that temporally, spatially, and semantically congruent information represented in more than one modality facilitates performance i.e. multimodal stimuli are detected faster or more accurately than incongruent bimodal stimuli [39]. This effect is not limited to one additional modality; Hecht et al. [41], for example, found that reaction times for tri-modal signals (visual, audio, haptic) were much faster than reaction times for bi-modal signals, which were in turn faster than uni-modal signals in a simple detection task. They note that although haptic perception was shown to be inferior during active movement, its combination with other modalities (visual and audio) is advantageous. Meyer et al. [11] showed that training with additional task-relevant visual and auditory cues can lead to transferrable improvements in performance.

The benefits of multisensory stimulation have been demonstrated in interface design studies in relation to improved task performance [42], user satisfaction [43] and reduced cognitive load [44, 45]. Tasks were generally performed faster, with greater efficiency, even when task difficulty was increased. For example, when assessing cognitive workload during the use of multimodal and unimodal interfaces, Shi et al. [44] and Oviatt et al. [45] showed that multimodal interfaces lead to reductions in cognitive workload reflected in objective (physiological) measures.

In 3D virtual interactions, there are limited possibilities to present haptic feedback: Force feedback conveys information to the user by the generation of forces using a mechanical interface that moves with the user and applies force to a specific point of the body, such as the finger-tip (via phantom haptic devices, [46]). As a substitute cue, vibrotactile feedback is a relatively simple mechanism for providing tactile signals [15], even though mechanical resistance to movements cannot be provided in this way [14–16]. Martinez et al. [14] compared force and vibrotactile feedback in a texture detection study and showed that vibrotactile feedback is more efficient than force feedback in the recognition of textures from different patterns and shapes. Similarly, Schoonmaker and Cao [16] suggested that the distortions in force feedback devices used during minimal invasive surgery make the task more difficult for surgeons and argue that vibrotactile feedback can serve as a viable substitute for force feedback. The empirical research investigating the benefits of vibrotactile feedback has shown potential advantages of vibrotactile feedback during VR interaction [2–4, 17–21, 26], spatial guidance [22], in teaching and learning processes of new physical activities [23, 24]. Vibrotactile feedback has also been shown to be an efficient warning signal in complex tasks such as driving a car or air traffic control [25].

Despite the supportive evidence, other studies have reported that sensory modalities, in particular tactile and audio feedback, can decrease overall performance; for example, audio and tactile cues were shown to have adverse effects in accuracy tasks, both were perceived as distracting and annoying and had a potential to cause sensory overload [26, 47, 48].

Sensory substitution has been applied successfully in helping people with deficits in one or more sensory modality [27]. Multimodal sensory substitution devices (SSDs) that provide audio and vibration cues to signal depth within the environment can successfully supplement visual information for low sighted and blind people [49]. Similarly, D’Alonzo and Cipriani [50] showed that vibrotactile sensory substitution can be used to induce self-attribution of the rubber hand during synchronous but modality conflicting visual tactile stimulation. In this study we provide additional substitute sensory cuing that is relevant to the manual task in a 3D environment.

### Presence and immersion

The principal aim in designing VR systems is to immerse users to such an extent in the virtual worlds that they accept the virtual world as ‘real’. The effectiveness of VR system is consequently often considered through the constructs known as ‘presence’ and ‘immersion’ [5–7, 29–32, 51–56]. The terms ‘presence’ and ‘immersion’ have been subject to debate within the research community [5–7, 29–32, 51–56]. There is a broad consensus that the term ‘presence’ describes the subjective feeling of being present in the virtual environment, rather than the real space. The term ‘presence’ has been defined as i) sense of being in one place or environment, even when one is physically situated in another [29–31, 34], ii) a state of consciousness, the (psychological) sense of being in the virtual environment [5–7]; iii) the sense of being physically present within a computer-generated or remote environment [32]; iv) user’s subjective, context-dependent psychological response to a VR system [13].

The definition of immersion has been subjected to a more controversial debate within the research community. Immersion is defined by some researchers, notably Witmer and Singer [29–31], as a subjective experience: the psychological state where one perceives himself as being included in and interacting with an environment that provides a continuous stream of stimuli and experience [29–32, 34, 55]. Other researchers, notably Slater [5–7], defined the term ‘immersion’ as a technological aspect of virtual environment i.e. the objective level of sensory fidelity a VR system provides. This is measurable in physical system parameters, such as the field of regard, field of view, stereoscopy, display size, display resolution, head based rendering, frame rate and refresh rate amongst others [5–7, 13, 33].

A variety of measures have been proposed to measure presence and immersion. The most common subjective measures are questionnaires (for a review see [56]). Objective measures include physiological measures [44, 45], time recordings [11, 39], eye movement [57] or task switching performance [58]. Visually evoked postural responses have been also proposed as an objective measure for presence in VR environment [21].

To assess user perception of the VR environment in this study, a short five-item questionnaire was used repeatedly; the items on this questionnaire were derived from the Presence Questionnaire (PQ) designed by Witmer and Singer [29–31]. The PQ questionnaire is frequently used to measure user perception of VR environment because of its convenience and non-intrusiveness [29–31, 53, 56, 59, 60]. PQ ratings are commonly found to the correlated with technological aspects of VR system design [61], performance and immersive tendencies of the user [53, 61] and the environment [56, 59, 60]. Empirical research has shown that VR technologies that produce a greater sense of immersion and involvement will also produce a higher sense of presence [29–34, 51–55]. Increased simulation realism (fidelity) is an important aspect of this and has been linked to enhanced presence and immersion, and consequently to improved performance [13, 32, 60, 62]. Some studies have shown that multimodal sensory input not only enhances the sense of presence and task performance, but also memory of objects in the VR environment [63, 64].

Witmer and Singer [29–31] suggest that presence in virtual environments (VE) depends on one’s attention shifting from physical environment to virtual one; how sharply users focus their attention on the VE partially determines the extent to which they become involved and how much presence they report [29–31]. Similarly, they suggest that immersion depends on one perceiving oneself as being part of the environment and being able to interact with it. Following this notion, presence would occur as a consequence of the allocation of attentional resources; immersion and involvement [29–31].

Slater’s definition of immersion as’ the degree to which VR technology delivers an objectively measurable match to real-world sensory signals’ [5–7] focuses on sensory fidelity [9]. In the experiments reported here, sensory fidelity is deliberately reduced by presenting increased levels of substitute cues, which provide task-relevant information. As described in the previous section, sensory substitution enhances performance in many ‘real environment’ applications and is likely to enhance subjective immersion in VR environment by providing more task-relevant cues that enable participant to interact with the virtual environment [29–31]. In this paper, we therefore follow definitions of presence and immersion as suggested by Witmer and colleagues [29–31]. It has been shown that the PQ measure, designed by Witmer and colleagues [29–31], addresses issues related to the level of involvement and control, the naturalness of the interaction, and the quality of the interface [29–31, 61, 65]. All of these aspects directly relate to our hypothesis which is focused on the evaluation of substitute sensory cues in VR environment during task performance, and as such this measure was seen as an appropriate baseline measure for our short version of presence questionnaire.

### The levels of fidelity in VR environment

It is generally assumed that high fidelity VR technologies are associated with better performance [13, 62]. At the same time, these technologies are often associated with a very high cost relative to their benefits [13, 52, 66, 67]. In the research community ‘fidelity’ is understood as a degree of similarity between real and simulated environment [52, 53, 60, 62–64, 66, 67]. Empirical research investigating the quantifiable benefits of high fidelity VR technologies suggests that rather than enhancing the overall VR set up, the enhancement of one or more individual components in VR can also be beneficial to performance [13, 52, 65, 66, 67]. This implies that the overall fidelity of the virtual environment may not be as important factor for overall performance. For example, McMahan et al. [66] provided empirical evidence for the benefits of using high fidelity VR technologies, but found that object manipulation can be successfully performed with lower fidelity VR technologies, such as less costly displays, with no loss of efficiency. Similarly, Dahlstrom et al. [67] found that a high level of simulator fidelity has little or no effects on skill transfer. They suggested that lower fidelity simulation can reduce complexity and enhance focus on training and should be used to complement higher fidelity simulations.

### Summary of experimental aims

In this study, task-relevant multisensory information is provided to represent cues that are not available in conventional VR simulations. The term ‘substitute cues’ is used to describe these sensory signals. This information may be presented in alternative modalities and may use cues that would not occur in real environments. We examine whether the available substituting sensory information improves task performance and users’ subjective experience, in particular their sense of presence in VR environment [5–7, 29–31, 51–56]. We hypothesize that substitute sensory cues will not be perceived as distraction; instead that substitute cueing will facilitate task performance and overall subjective experience of the users. We examine which sensory cues (audio, tactile, visual) are most effective for objective and subjective performance measures. Additionally, we also examine the role of modes of feedback (multisensory, bimodal, unimodal) on users performance. Furthermore, we establish whether a relationship between the users’ subjective experience and overall objective task performance exists.

## Methods

### Participants

The current study was approved by the University of Liverpool's Institute of Psychology and Health Sciences Ethics Committee (PSYC-1112–049A). The individual portrayed in one of the pictures in this manuscript and the individual appearing in the video (S1 Video) have given their written informed consent (as outlined in PLOS consent form) to publish these case details. Seventeen participants were recruited via opportunity sampling (12 male, 5 female, aged between 18 and 48, M = 26.7, SD = 12.4). All participants gave informed consent and reported normal or corrected-to-normal vision and normal hearing.

### Apparatus

The experiment was conducted at the Virtual Engineering Centre (VEC) facility located in the Science and Technologies Facilities Council (STFC) in Daresbury, UK. The task was to interact within the virtual environment by holding a pneumatic tool (impact wrench) and perform a wheel change on a virtual racing car whilst substitute cues, presented in the form of visual, tactile and audio sensory feedback, provided additional task-relevant information.

### Virtual reality set up

**Fig 1:**
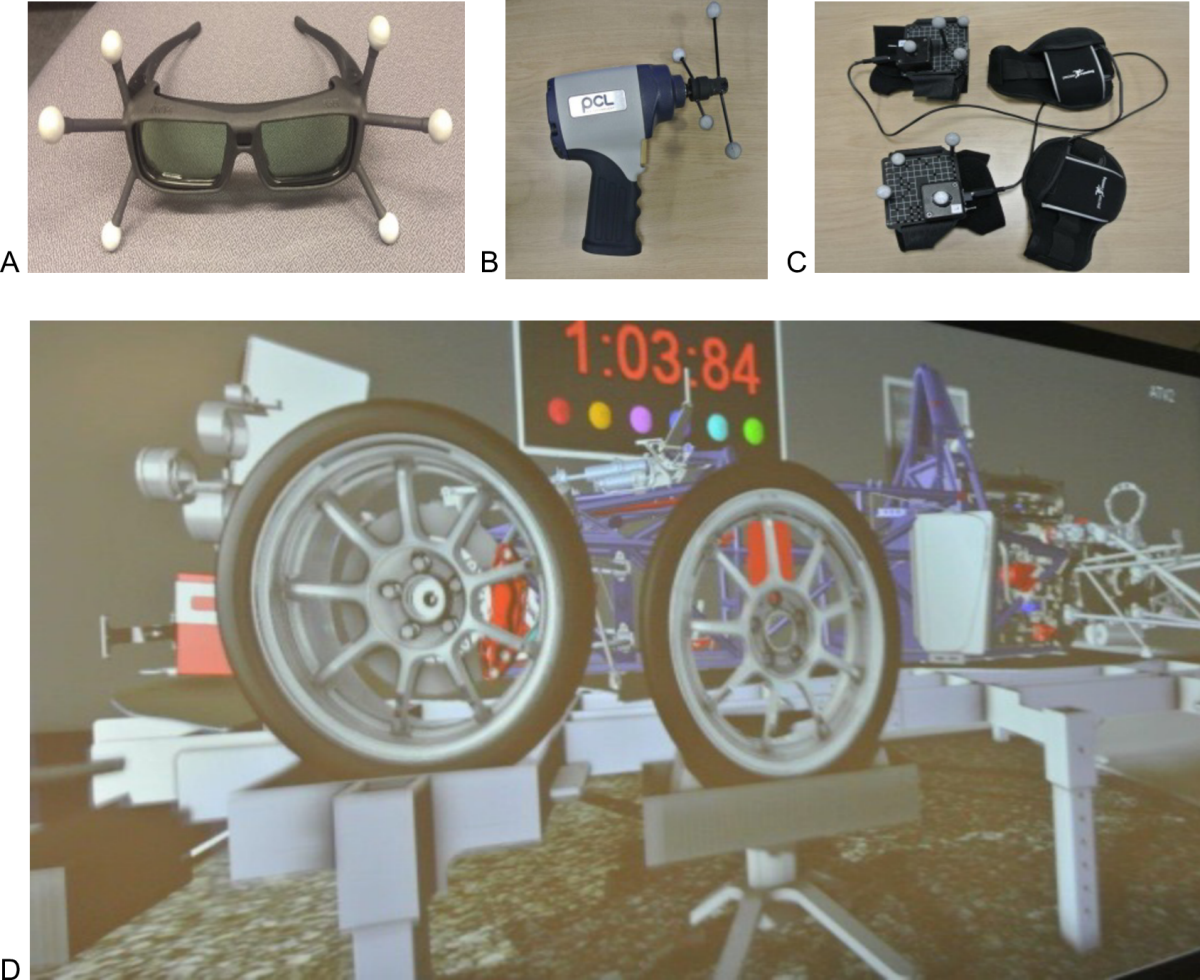
Virtual tools and set up. Apparatus used in the experiment: (A) 3D shutter glasses, (B) impact wrench and (C) haptic gloves. Picture D shows the position of the wheel when the task is completed (first wheel on the stand, second wheel on the racing car).

The virtual reality set up consisted of a planar display screen 6m in length and 2.1m in height, behind which were two active stereo projectors that create 3390 x 1200 resolution images at a rate of 120Hz. 3D stereo images were produced using an NVIDIA Quadro K6000 GPU. Participants wore wireless LCD shutter glasses that were synchronized with the projectors to provide stereoscopic images. 16 high-spec infrared cameras (VICON Bonita B10 with 250 fps capture speed, motion resolution of 0.5mm of translation and 0.5 degrees of rotation in a 4m x 4m volume using 9mm markers) were used to track object motion in the VR environment. Position data, computed using VICON Tracker software, was broadcast in real-time across the internal network using a VRPN protocol at a rate of 200Hz and used to update the virtual environment. The following objects were tracked in order to provide the required interaction within the virtual immersive environment: LCD shutter glasses (for head tracking and POV adjustment), haptic gloves on subject’s hands (to drive the subjects’ virtual hands) and the impact wrench (PLC Prestige 1/2”, weight = 1.3kg, 15.2cm long), the tool used to remove the bolts from the wheel (Fig 1).

**Fig 2:**
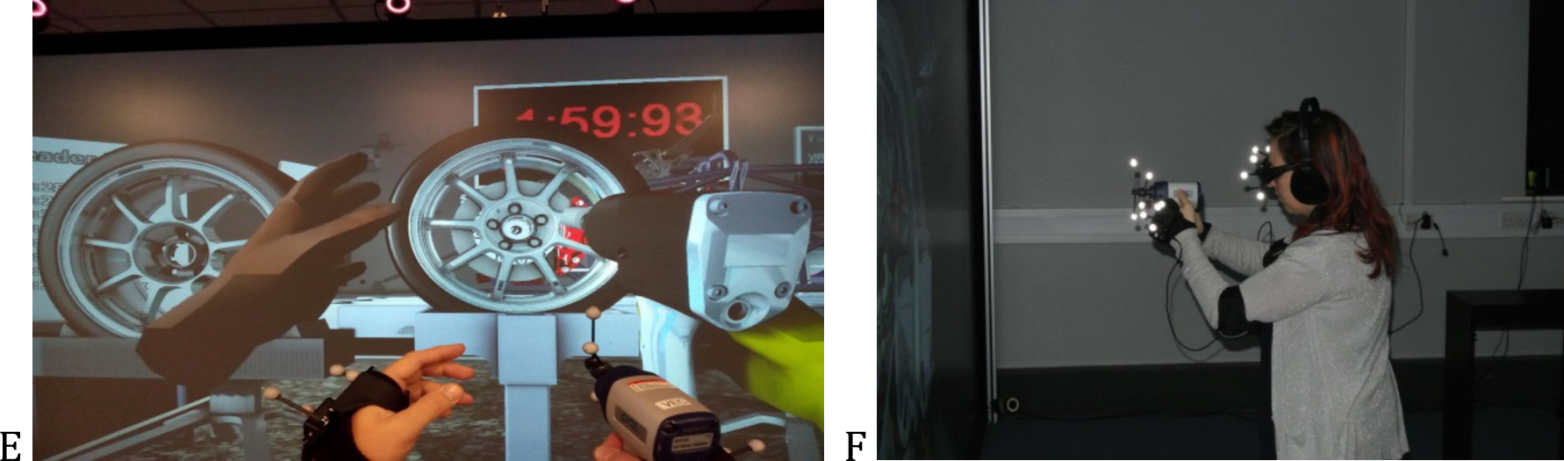
View during the task. The simulation view from a participants’ perspective: (E) Participants hand and tool were co-located with their virtual counterparts i.e. participants were not able to see virtual hands or tool (F) Participant wore 3D shutter glasses, headphones, vibration gloves and were holding impact wrench whilst performing the task.

The wheel change simulation was designed using the 3DVia system running at a constant speed of 75fps across all possible combinations of cues to ensure an accurate time recording in all experimental conditions. The virtual simulation consisted of a virtual racing car that was positioned on a stand in the middle of the screen. The virtual scene also contained two stands that are positioned 68cm in x direction, 14cm in y direction and 8cm in z direction from the centre point of the wheel on the virtual car. The stand on the right side of the car held the spare tyre and the stand on the left side of the car was free for participants to put the car tyre on. In order to pick the wheel up participants had to stand directly in front of the wheel, thus every participant had to make similar postural adjustments to get in contact with the wheel. A faithful digital mock-up of the impact wrench was used to interact with the bolts. Through accurate calibration, both hands and the impact wrench overlapped with their virtual counterparts from the participant’s perspective (Fig 2).

Tactile feedback was provided by “tactile gloves” which had vibration motors attached to the back of the palm of each of the VICON hand tracking kits. The tactile gloves provided variable vibration cues, ranging from 15Hz (when participants hold the bolt) to 250Hz (the strongest vibration experienced when bolt is in), via a wireless interface with the simulation CPU. The vibration cues were designed to mimic the intensity of vibration generated by the impact wrench when performing the wheel change task. For example, the subject felt an intermediate level of vibration when screwing a bolt out or back in place (85Hz), which increased to the maximum level (250Hz) as soon as the bolt is completely screwed in, or reduced to zero when it is completely removed.

Audio feedback was presented via SONY headphones (RF811RK Wireless Headphones) with the frequency responses from 20 to 20 kHz. When audio cues were enabled they provided task-appropriate sounds, such as sampled recording of a torque wrench. When audio cues were disabled, a white noise was played to mask the sound generated by the vibration motors in the haptic gloves.

### Task procedure

The experiment was conducted in a dedicated VR laboratory with no distraction, which is consistent with previous research where participants are engaged in their activity but not aware of the observers or other distractions [34]. Participants were explained the main definitions of the constructs that are being evaluated during the study. For the task, participants wore 3D shutter glasses, vibration gloves and headphones that played either audio cues (when they were on) or a continuous white noise to mask any vibration noise from the haptic gloves (Fig 2). The task was to change the wheel on the virtual racing car in the 3D environment as fast as possible. The substitute sensory cues were presented as unimodal, bimodal and multimodal feedback in a counterbalanced quasi-random order. Every participant started with two practice trials.

The time started when participants got in contact with the physical tool (impact wrench). First, they had to unscrew five bolts from the wheel on the virtual racing car. Then they had to put down the impact wrench, pick up the wheel and place it on the stand located on the right side of the racing car axle (distance 68cm). After this, they had to go and take the replacement wheel from the stand on the left side of the racing car, attach it on the racing car, take hold of the impact wrench and screw the bolts back in. The overall recording stopped when the participants placed the tool back on the table, which was located on the right side, approximately 1.5m from the projection display. Between each condition, participants’ subjective perceptions of the VR environment were recorded on a short questionnaire adapted from PQ measure [29–31]. The video recording of the whole task can be found in S1 Video.

### Multisensory feedback cues

A projection-based VR system, where visual information is always present, was used in this study. The substitute cues (visual, tactile, audio) provided additional information to compensate for other cues that are not readily available in VR environments, such as weight or torque.

Substitute visual cues consisted of bolts turning yellow when in contact with the tool and red when the bolts are completely in or out; the wheel turning yellow when in contact and red when in the correct position; the virtual hands of the participant turning yellow when in contact with virtual objects. Tactile cues were presented as vibration when: the tool was in contact with the bolt and a more intense vibration when the bolt was completely in or out; when the virtual hands were in contact with the wheel (carrying the wheel from the car to the stand and from the stand on to the car). Audio cues included a sampled impact wrench noise that was played when the virtual wrench was in contact with the bolt and a ‘snap’ sound when the wheel was placed on the stand and on the car.

### Objective and subjective performance measures

In this study overall task performance was used as an objective measure. Participants were instructed to perform the task as fast as they can. The mean completion times for each condition were used in the statistical analysis. As an incentive, a leader board was displayed within the virtual scene where the participants’ fastest times were shown and updated after each participant had completed the task.

In order to investigate participants’ experience, participants were required to fill in a short questionnaire after each experimental condition. The selected dimension for the presence measure was a continuous scale. The questionnaire contained seven items measuring the sense of involvement and immersion (5 questions), enjoyment (1 question) and general discomfort (1 question). The questions concerning the perceived sense of involvement and immersion were adapted from Presence Questionnaire (PQ) developed by Witmer and Singer [29–31] to collectively capture the ‘the sense of being present inside’ the VR environment. All adapted questions were transformed into statements and participants were asked to mark on the line (0-not at all, to 10-completely) how strongly they agree or disagree with the statements after each sensory condition. Higher responses on the questions concerning the levels of involvement and immersion indicated an increased sense of presence [29–31]. The statements were as follows:

The interaction with the environment seems natural (control factor);

I felt that sensory cues helped me in completing the assigned task (sensory factor);

The sense of immersion inside the virtual environment was compelling (realism/immersion factor);

The virtual environment felt real (realism/ immersion factor);

The virtual environment was responsive to my actions (distraction factor).

To capture immediate attitudes about presented sensory cues two additional statements were included:

Doing the task felt enjoyable (enjoyment);

I experienced general discomfort (discomfort).

Although no questions directly addressed the state of ‘being in the virtual environment’ we assume that they collectively measure the construct known as ‘presence’, as the questions were adopted from a 4-factor model of presence derived from the factor analysis of the Presence Questionnaire (PQ) [29–31]. Reliability analysis of the questionnaire is reported in the results section.

### Experimental design

This study adopted a repeated measures within-subject factorial (2x2x2, presentation of substitute audio x tactile x visual) design. A single group of participants performed the task in all conditions in a quasi-random sequence. There were eight possible combinations of sensory feedback provided: audio (A), visual (V), tactile (T), audio-visual (AV), audio-tactile (AT), tactile-visual (TV) and audio-visual-tactile (AVT) as well as condition where no additional cues were presented (NONE). The task completion times were logarithmically transformed to satisfy the normality assumption (Shapiro-Wilk) for statistical analysis. An analysis of variance (ANOVA) was performed on mean completion times to investigate the effects of each sensory modality: visual, audio and tactile cues were set as binary factors that were either present or absent. The Mauchly test of sphericity was applied and when significant, Greenhouse-Geisser corrections were adopted. Partial eta squared is reported for effect sizes. Paired sample t tests, with correction for multiple comparisons (Sidak), were conducted to investigate whether the availability of sensory modality affected participants’ behaviour and performance. When the direction of the relationship was predicted, one tailed test results were reported. The significance level for all statistical tests was set at 0.05. For paired sample t tests, Cohen’s d [68] was chosen as a measure of effect size, which is calculated as the difference between two means divided by pooled standard deviation. The accepted suggestions for the magnitude of effect sizes are: 0.2 = small effect, 0.3 = medium effect and 0.5 = large effect [68].

## Results

### Objective measures

**Fig 3:**
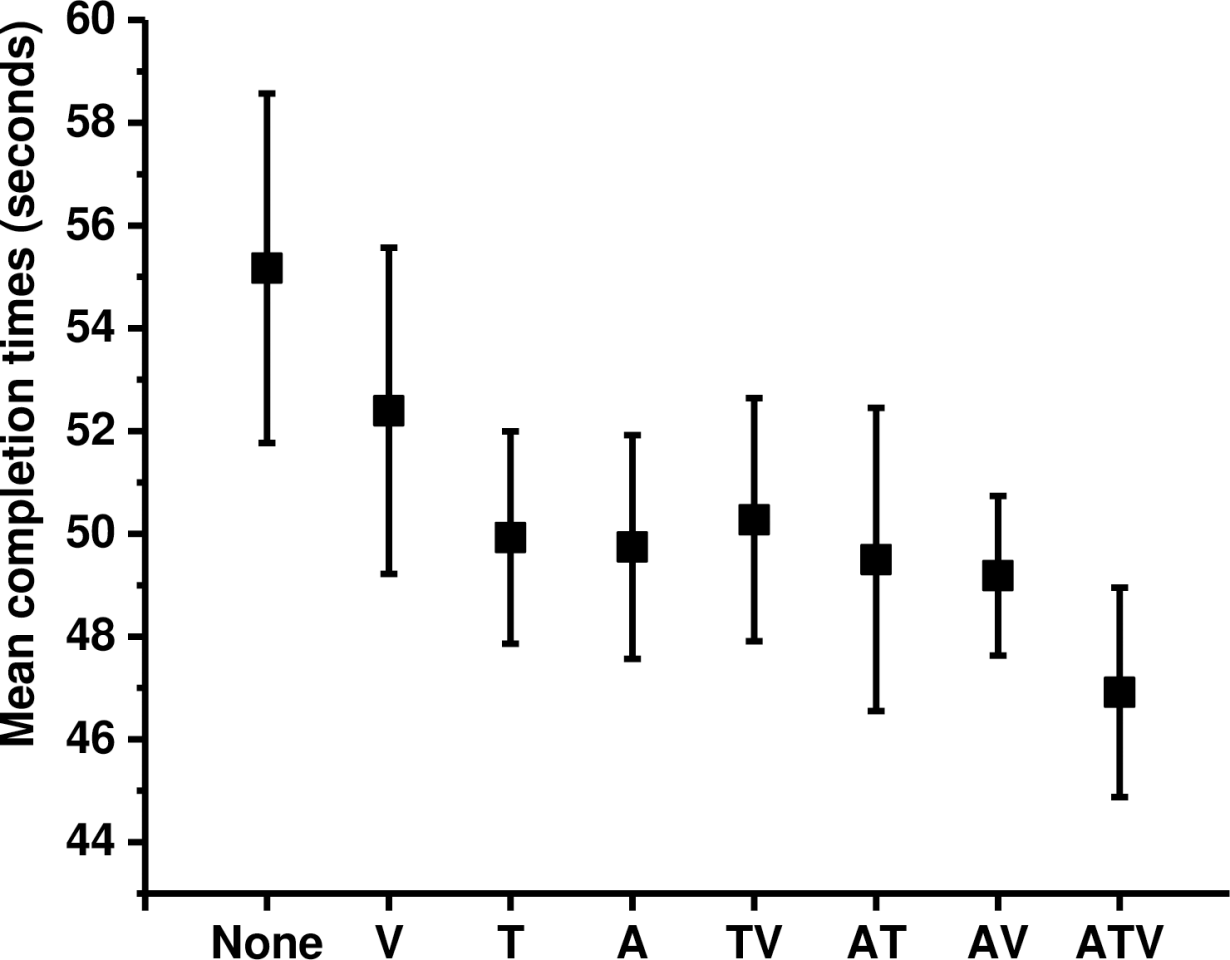
Objective performance data. Means and standard errors in each condition for mean completion times. Participants were asked to perform the task as fast as they can; therefore shorter completion times indicate better performance. The data indicate that as the amount of sensory cues in the simulation increased, the task was completed faster.

It took participants 50.3 seconds (SE = 1.9) to complete the virtual wheel change task on average. The mean task completion times in each sensory cue condition are shown on Fig 3. The order of conditions that facilitated performance the most is: ATV (M = 46.9, SE = 2.03), AV (M = 49.1, SE = 1.54), AT (M = 49.5, SE = 2.9), TV (M = 50.2, SE = 2.36), A (M = 49.7, SE = 2.17), T (M = 49.9, SE = 2.06), V (M = 52.3, SE = 3.17) and NONE (M = 55.1, SE = 3.4).

**Fig 4:**
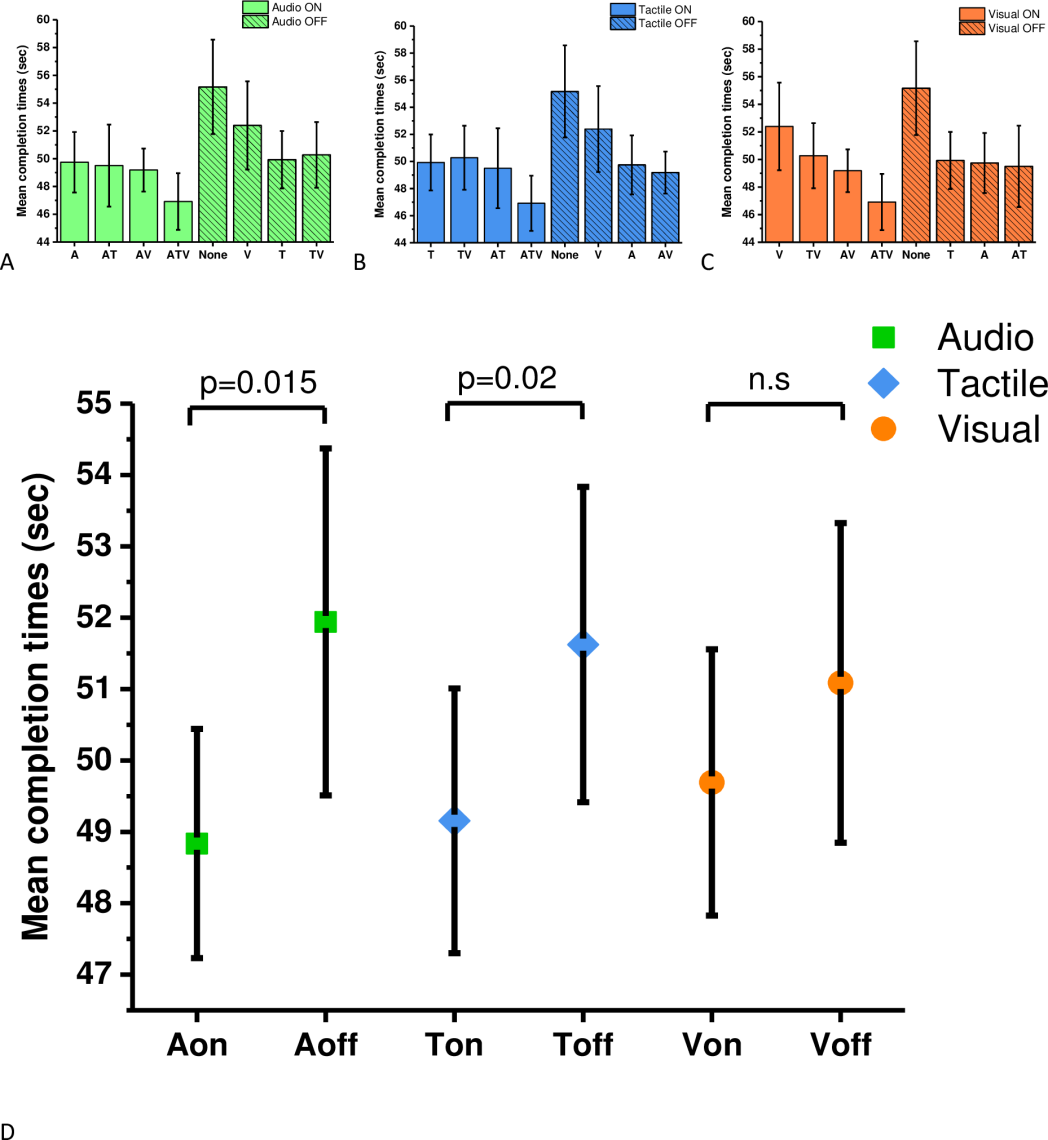
Performance data for each sensory cue. To visualise the data easily, all sensory cues were separated into groups when each of the cues were present or absent. The graphs display the mean completion times and standard errors for (A) audio, (B) tactile and (C) visual cues. Paired sample t-tests with Sidak correction revealed a significant effect of audio (p = 0.015) and tactile cues (p = 0.025). No significant differences were found for visual cues (p = 0.15).

A (2x2x2) repeated measures ANOVA revealed that there were significant main effects in objective performance caused by the presentation of audio (F(1,16) = 5.87, p = 0.028, η^2^ = 0.27) and tactile cues (F(1,16) = 4.48, p = 0.043, η^2^ = 0.24), but not for additional visual cues (F(1,16) = 0.433, p = 0.52, η^2^ = 0.03). No significant interactions between modalities were observed. Planned comparisons using Sidak adjustment (p < 0.02) were performed on data that were separated into groups where each sensory modality was either present or absent. The analysis revealed that the task was performed significantly faster when audio cues were present (M = 48.8, SE = 1.61; t(16) = -2.324, p = 0.015, d = 0.36, one tailed) compared to conditions when white noise was played (M = 51.9, SE = 2.42). Significant differences were also observed for tactile modality; the mean time to complete the task was significantly faster when the tactile cues were present (M = 49.6, SE = 1.85; t(16) = -2.044, p = 0.025, d = 0.29, one tailed) as opposed to trials when tactile cues were absent (M = 51.6, SE = 2.21). No significant difference was observed on mean completion times for additional information bearing visual cues (M = 49.6, SE = 1.86; t(16) = -1.027, p = 0.15, d = 0.17, one tailed) vs (M = 51.1, SE = 2.23) (Fig 4).

**Fig 5:**
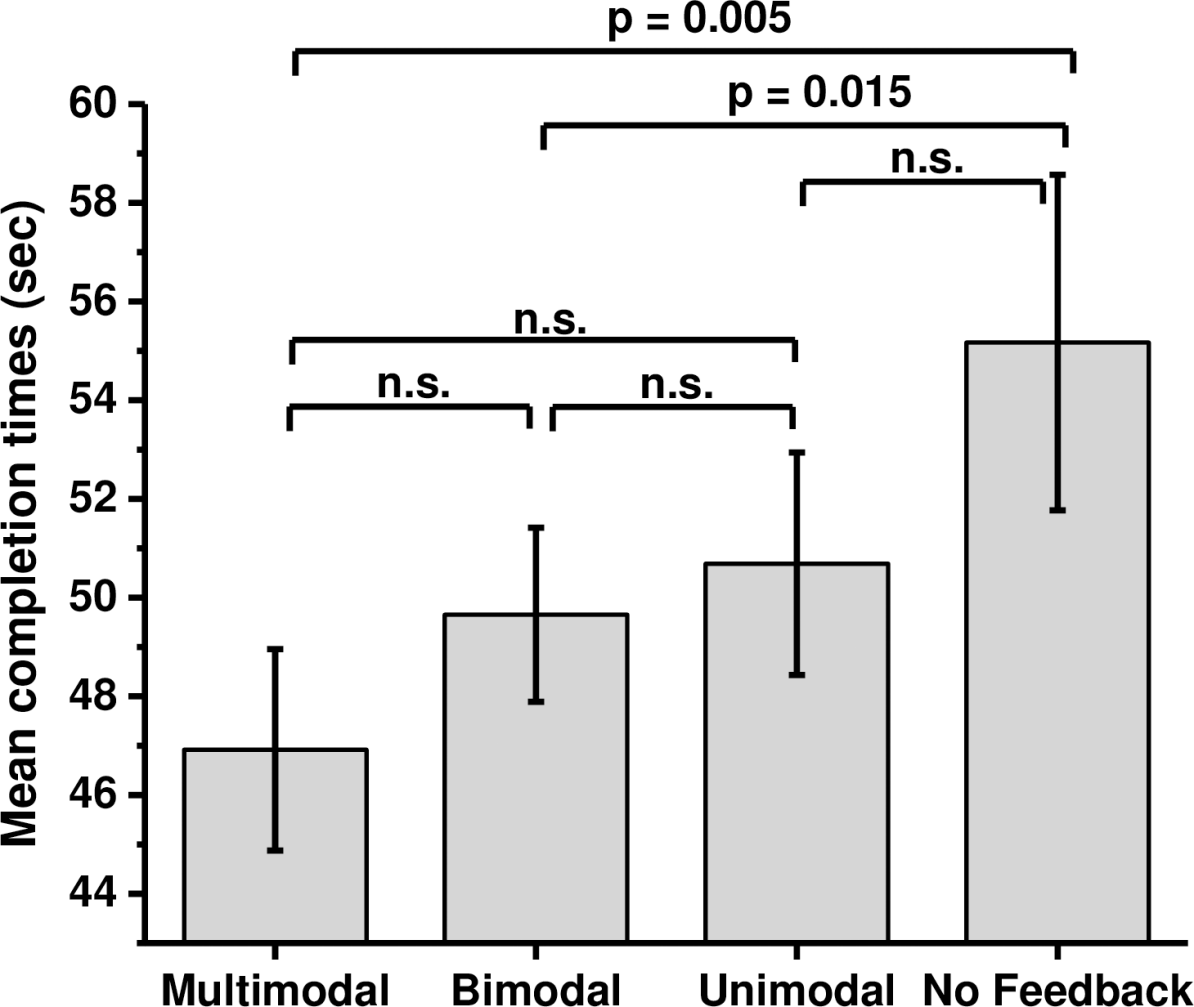
Effect of feedback modes on objective data. Means and standard errors of overall completion times for each mode of feedback: multimodal, bimodal, unimodal, and no feedback. Data for bimodal (AV, AT, TV) and unimodal (A, T, V) category were group together and the mean scores were used for the analysis. A significant difference was observed between the multimodal and no feedback condition (p = 0.005) and the difference between no feedback and the bimodal feedback was significance after adjustment (p = 0.017).

To further investigate which form of sensory feedback was most beneficial to the overall task performance, pairwise comparisons on mean completion times in each mode of feedback were conducted. The data were grouped into four modes of feedback (unimodal, bimodal, multimodal and no feedback) and the means across the groups were used for the analysis (Fig 5). Repeated measures ANOVA with Greenhouse-Geisser correction revealed a significant effect of mode of feedback (F(3,48) = 5.164, p = 0.004, η^2^ = 0.24). Planned comparisons with Sidak adjustment (p < 0.01) revealed that the task was performed significantly faster with multimodal feedback (M = 46.9, SE = 2.04) as opposed to no feedback (M = 55.2, SE = 3.4; t(16) = -2.884, p = 0.005, d = 0.7). There was no significant difference between multimodal feedback (M = 46.9, SE = 2.04) and unimodal feedback (M = 50.7, SE = 2.26, p = 0.02, d = 0.5) or bimodal feedback (M = 49.7, SE = 1.8; p = 0.03, d = 0.47). The difference in mean times between the no feedback condition (M = 55.2, SE = 3.4) and bimodal feedback (M = 49.7, SE = 1.8) was recorded significant after adjustment (t (16) = -2.361, p = 0.015, d = 0.57); no significance was obtained with unimodal feedback (M = 50.7, SE = 2.26; p = 0.03, d = 0.48) or between bimodal (M = 49.7, SE = 1.8) and unimodal feedback (M = 50.7, SE = 2.26; p = 0.25, d = 0.16).

### Subjective measures

**Fig 6:**
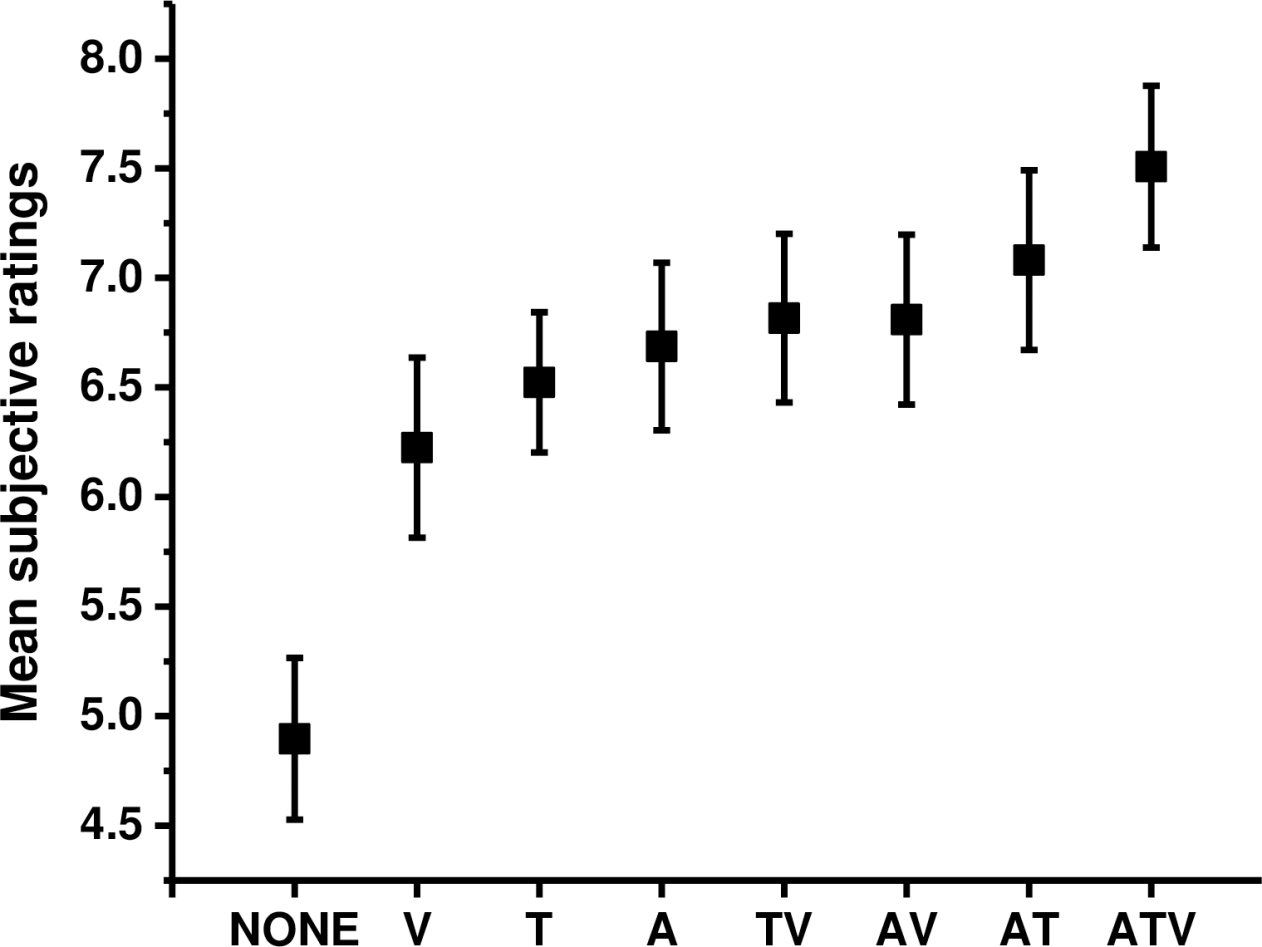
Subjective rating scores. The mean subjective ratings and standard errors of presence ratings in all experimental conditions. Participants were asked to rate on a continuous scale from 0 (not at all) to 10 (completely) how strongly they agree or disagree with the statement. All questions were formulated in a positive manner, thus higher scores indicate an enhanced sense of presence.

In order to assess user acceptability of the virtual environment, participants were asked to rate their sense of involvement and immersion using a short questionnaire after each experimental condition. The questions were adapted from a 4-factor model of presence, proposed by Witmer and colleagues [29–31] who suggested that both involvement and immersion are necessary to experience an increased level of presence. The reliability of the rating scale used in this study was assessed by calculating the Cronbach coefficient—alpha [55]. The standardised alpha of rating scales showed acceptable reliability (sense of presence = .97). The overall mean rating score was 6.65 (SE = 1.74). The mean subjective ratings in each condition can be seen on Fig 6. The order of conditions that were perceived as the most compelling is as follows: ATV (M = 7.51, SE = 0.37), AT (M = 7.08, SE = 0.41), AV (M = 6.81, SE = 0.38), TV (M = 6.81, SE = 0.38), A (M = 6.68, SE = 0.38), T (M = 6.52, SE = 0.31), V (M = 6.22, SE = 0.41) and NONE (M = 4.91, SE = 0.36).

**Fig 7:**
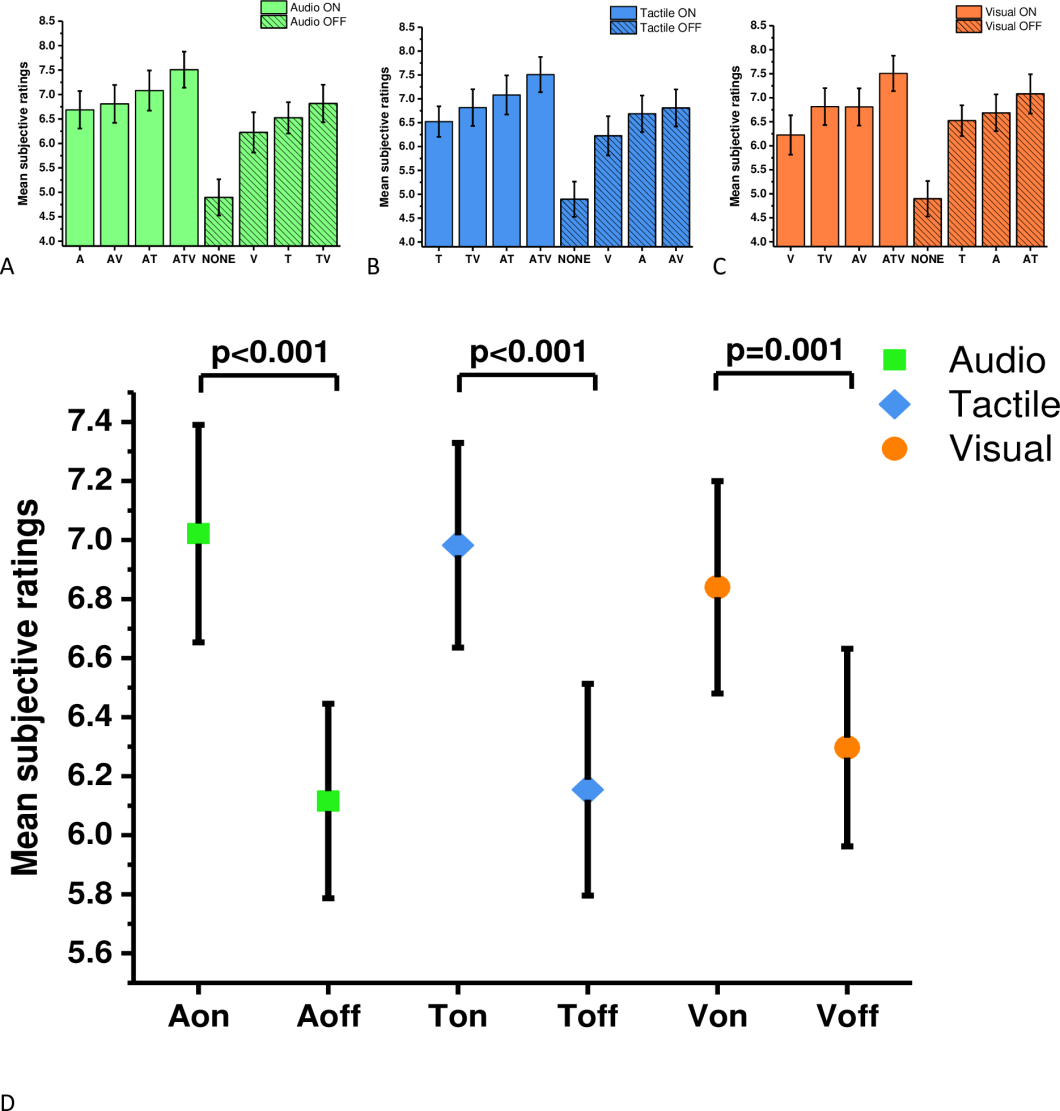
Subjective ratings for each sensory cue. The means and standard errors for subjective ratings of presence: (A) audio, (B) tactile and (C) visual cues. Panel A, B and C shows the same data as panel D, however they are in a different order to aid a visual comparison. Significant effects in all three sensory modalities were found (D).

The analysis of the subjective data was performed analogous to the analysis of the objective data. The analysis with Greenhouse-Greissler correction determined that all sensory modalities significantly influenced subjective ratings: there was a main effect of audio modality (F(1,16) = 33.45, p < 0.001, η^2^ = 0.68), a main effect of tactile modality (F(1,16) = 20.34, p < 0.001, η^2^ = 0.56) and a main effect of visual modality (F(1,16) = 15.74, p = 0.001, η^2^ = 0.5). Significant interactions between sensory modalities were also recorded between audio and tactile (F(1,16) = 5.19, p = 0.03, η^2^ = 0.25); audio and visual (F(1,16) = 6.34, p = 0.02, η^2^ = 0.28); tactile and visual (F(1,16) = 10.10, p = 0.006, η^2^ = 0.39) and audio, tactile and visual (F(1,16) = 15.07, p = 0.001, η^2^ = 0.49). Planned comparisons using Sidak adjustment (p < 0.01) were computed on mean scores where each modality was either present or absent (Fig 7). The results revealed that participants experienced a higher sense of presence when audio cues were present (M = 7.02, SE = 0.36) as opposed to absent (M = 6.11, SE = 0.32; t (16) = 5.774, p < 0.001, d = 1.39). The same effect was seen for tactile cues (M = 6.98, SE = 0.34) versus (M = 6.15, SE = 0.35; t(16) = 4.51, p < 0.001, d = 1.06)), and for visual cues (M = 6.84, SE = 0.35) versus (M = 6.29, SE = 0.33; t(16) = 3.961, p = 0.001, d = 1.03).

**Fig 8:**
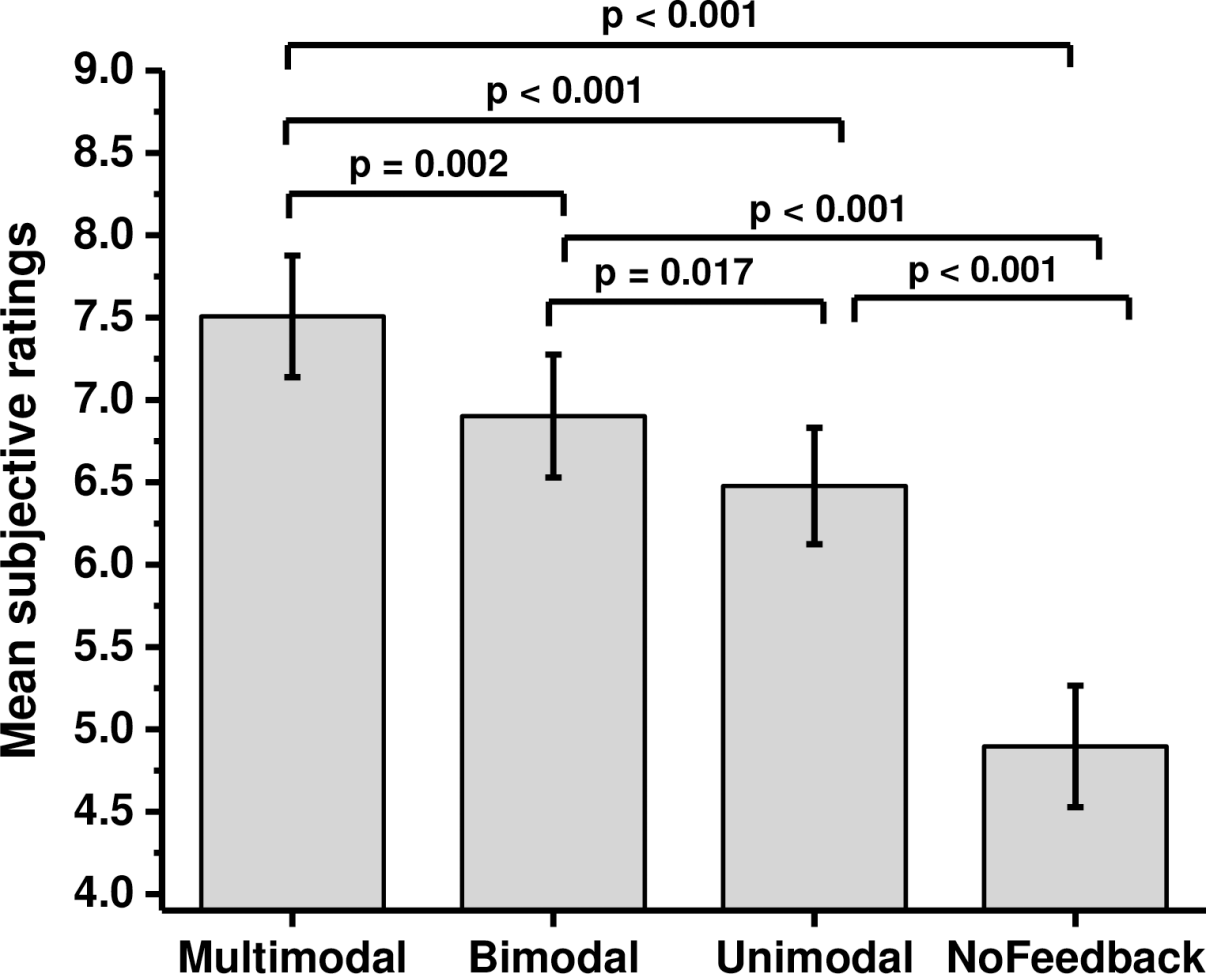
Effects of feedback modes on subjective ratings. The graph shows means and standard errors of subjective ratings in each mode of feedback.

The effects of different modes of feedback on subjective ratings were also examined (Fig 8). Repeated measures ANOVA revealed a significant effect of mode of feedback (F(3,48) = 37.350, p < 0.001, η^2^ = 0.7). Planned comparisons with Sidak adjustment (p < 0.01) revealed that the subjective ratings of presence were significantly higher when multimodal feedback was presented (M = 7.51, SE = 0.37) as compared to bimodal feedback (M = 6.91, SE = 0.37, t(16) = 3.794, p = 0.002, d = 0.86), unimodal feedback (M = 6.48, SE = 0.35; t(16) = 4.964, p < 0.001, d = 1.25) and no feedback (M = 4.89, SE = 0.37; t(16) = 7.210, p < 0.001, d = 1.79). Subjective ratings also differed significantly between no feedback (M = 4.89, SE = 0.37) and unimodal feedback conditions (M = 6.48, SE = 0.35; t(16) = 6.717, p < 0.001, d = 1.51) and between no feedback and bimodal feedback conditions (M = 6.91, SE = 0.37, t(16) = 5.809, p < 0.001, d = 1.44). The difference between bimodal (M = 6.91, SE = 0.37) and unimodal feedback conditions (M = 6.48, SE = 0.35) was also recorded significant (t(16) = 2.651, p = 0.017, d = 0.75).

**Fig 9:**
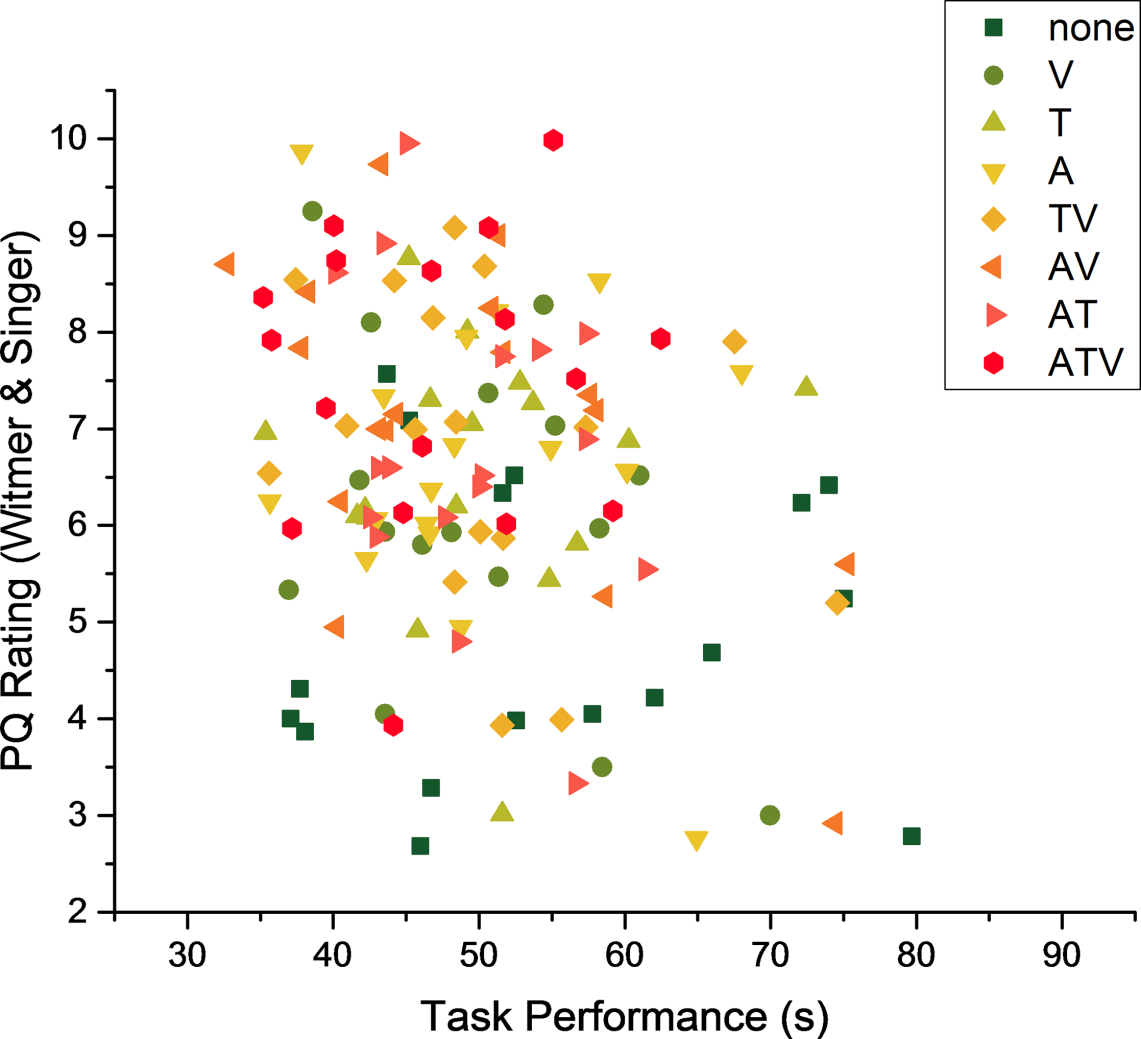
Objective and subjective data for all participants in every sensory condition. Reddish colour indicates that as the amount of sensory cues increased (red and orange colours) the overall performance improved.

The main focus of this study was to evaluate the contribution of substitute cues to the overall task performance and user experience. Previous research suggested that the experienced sense of involvement and immersion, collectively also referred to as the sense of presence [29–31], might impact user’s ability to perform a task [53, 60]. One of the goals of this study was to investigate whether the user’s subjective sense of presence, as measured by the questions addressing involvement and immersion, is related to their overall task performance. In order to investigate this relationship the Pearson correlation analyses were performed. The Fig 9 shows data points for all participants in all sensory conditions.

A fundamental problem when investigating relationship between (subjective) self-evaluation and (objective) performance data is in the intrinsic inter-individual variability that is present in the subjective data [69]. While (objective) performance is measured relative to an external and common standard (the time to complete the task), the subjective measure (for example presence ratings) relies on internal scales that are unique to each observer. This means that individual subjective ratings can only reflect the relative changes between conditions that are experienced by the users and that there is no common subjective standard or common range of responses across participants. Individuals can, however, reliably judge relative subjective changes in their experience across the conditions they experience.

**Fig 10:**
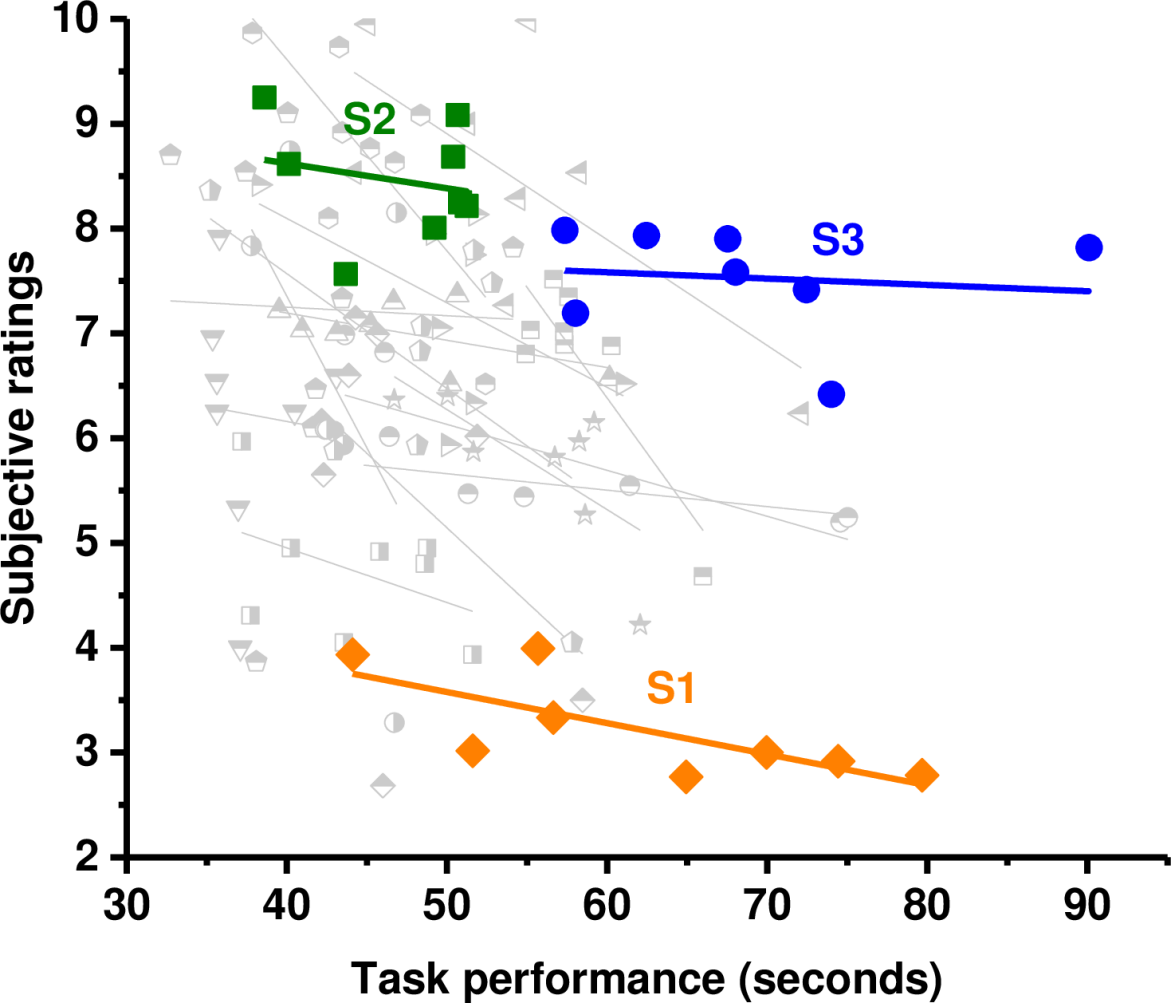
Correlations. Correlational analyses on individual data collected from each participant. Participant 1 (S1), participant 2 (S2) and participant 3 (S3) show comparable performance but highly idiosyncratic differences in their subjective ratings.

To illustrate this we selected data from three participants (Fig 10). The graph shows objective performance and subjective ratings for each of the eight conditions that were tested. A straight line fit shows the overall relationship between objective and subjective measure. This negative relationship between task completion time (in seconds) and presence judgements is seen for all participants. The subjective ratings however show significant idiosyncratic differences: participant 1 (S1, orange), for example, gave consistently low ratings for presence (around 3/10), while participant 2 (S2, green) and participant 3 (S3, blue) gave consistently much higher ratings (around 8/10) although the objective performance range was comparable. The subjective ratings for S2 and S3 are comparable although there is no overlap in their objective performance (S2 took at most 50 secs to complete the task while S3 took at least 55 secs). Participants have no access to a common standard for their subjective evaluation; it is therefore not surprising that there are significant variations in the mean ratings. While the objective conditions that each participant experiences are the same, there is no reason to expect that the range in objective differences between conditions will result in comparable ranges of subjective experience. All three subjects, in common with the majority of all of our subjects however, show a significant correlation between their individual subjective ratings and objective performance.

**Fig 11:**
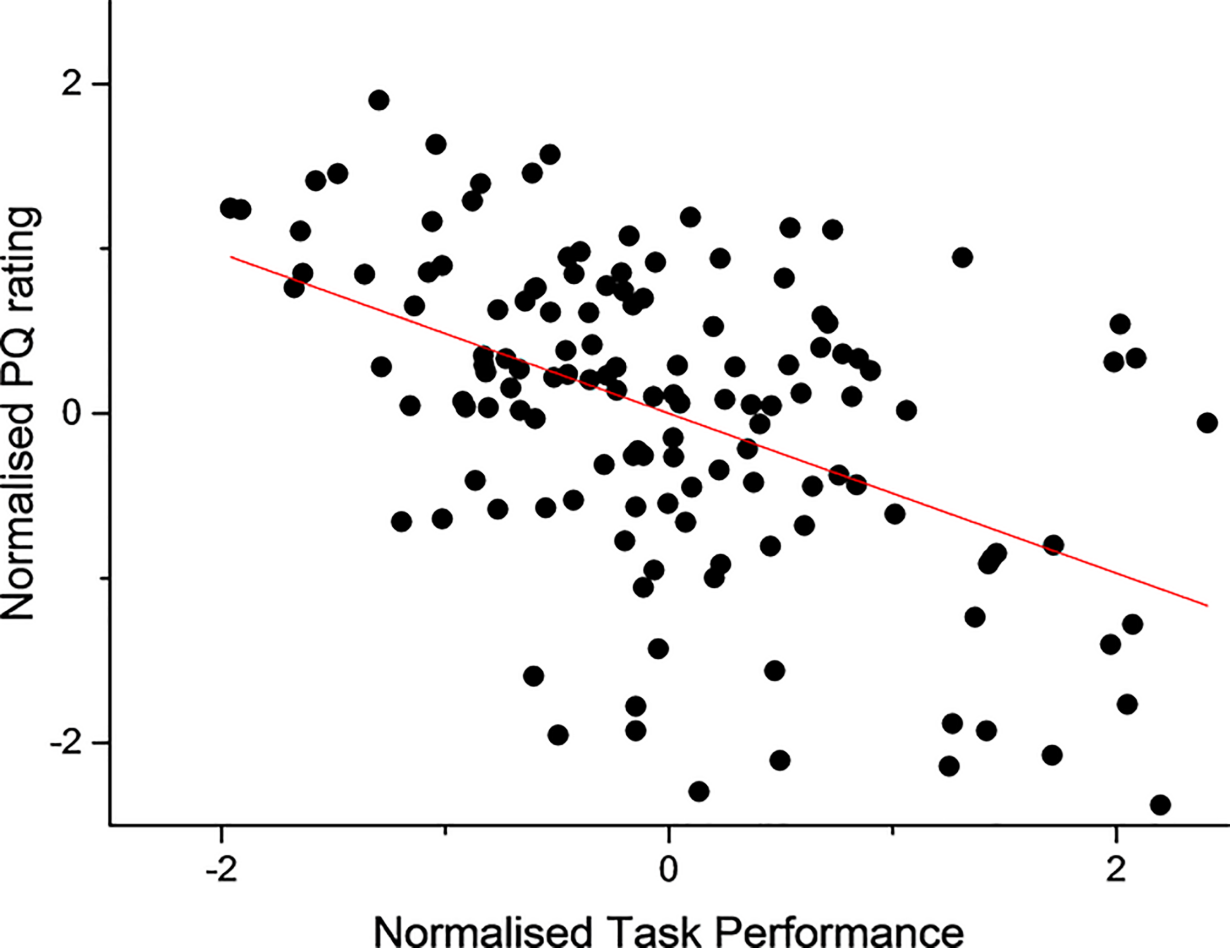
Correlation. The correlational analysis was performed on the normalised data across all participants in every sensory condition that was tested (r = -0.485, n = 17, p < 0.001).

To correlate the objective and subjective measures across the entire pool of participants the idiosyncratic differences between participants have to be resolved. We suggest that there are two ways to approach this: one approach is to normalise the individual raw data; a second approach is to correlate the pooled mean of the data across conditions. The correlations of the normalised data are presented here; correlations of the raw data and mean data (from each sensory condition) are provided in S1, S2, S3 and S4 Files. All objective and subjective data recorded in this study have been normalised so that every subject had same common mean (0) and same standard deviation (1). The results showed consistent negative regression lines between objective and subjective measures in every participant; for ten out of seventeen subjects the two measures were significantly correlated. The negative correlation implies that when participants experienced an increased sense of involvement and immersion, commonly referred to as the sense of presence [29–31], their overall task performance improved i.e. they performed the task faster. A correlation analysis across all participants and all conditions shows a strong significant negative correlation (r = -0.485, p < 0.001) between the performance measures and users’ perceived sense of involvement and immersion within the VR environment (Fig 11).

## Discussion

This study investigated how additional substitute multisensory cues affect human objective performance and subjective evaluation during the completion of a realistic task in a VR environment. During the wheel change task different combinations of substitute sensory cues that carried task-relevant information were provided in order to aid performance.

Our principal finding is that the time to complete the task, our objective performance measure, significantly reduces when additional information-bearing cues are presented. The subjective user experience, recorded through a short questionnaire adopted from previous research [29–31] also shows significant improvements in ratings. The substitute cues that were presented were information bearing cue, but not natural cue; they therefore have a negative impact on surface fidelity and, following this, might be expected to cause a reduction in the sense of presence probed in the subjective data. Our data shows that there is no direct positive relationship between surface fidelity and objective performance or subjective ratings in our task. A possible explanation is that the improvement in informational fidelity outweighs the loss in surface fidelity. Previous studies have shown that increasing the fidelity of various components of VR environment, rather than overall VR set up, could be beneficial to performance [13, 52, 66, 67]. Our findings support this research and further imply that some tasks in VR environment can be successfully performed in low fidelity VR environments with no loss on efficiency [14, 33].

The advantages of auditory and tactile feedback have been noted in previous research; for example, it was reported that audio feedback can improve task performance in terms of accuracy and spatial attention without affecting perceptual workload [11, 15, 16, 39, 40–45, 49, 50]. Similarly, haptic feedback was shown to improve interaction, spatial guidance and learning in VR environments [2–4, 23–25, 50]. The analysis of the objective measures in this study revealed significant effects of substitute cues that were presented in the audio and tactile modalities; however the effect of cue substitution in the visual modality did not reach significance. This finding may be explained by the visual information load [25, 38]: during the visual condition no meaningful audio or tactile signals were provided so that substitute cues increase the load in a single modality.

Previous research has reported some benefits of multimodal interfaces i.e. for spatial understanding [5], accuracy of performance [37, 39], in gaming environments [64] and enhanced performance in dynamic threat scenarios [14]. Similarly, in this study we show that multimodal feedback improved overall task performance as well as enhanced the levels of presence and immersion. Our data are consistent with previous findings suggesting that appropriate sensory cues can improve task performance [5–7, 14, 37, 39, 64]. One surprising finding stemming from this research was the effectiveness of audio and tactile feedback when presented unimodally. It has been noted that audio and tactile feedback can be perceived as distracting and annoying and can even decrease the accuracy of performance [20, 33]. However, in our study unimodal audio and tactile feedback influenced performance and subjective ratings positively. Individually, the mean time for audio condition was 49.7 seconds and for tactile it was 49.9 seconds. In comparison to bimodal conditions i.e. audio-visual (AV = 49.1s), audio-tactile (AT = 49.5s) and tactile-visual (TV = 50.2s) it can be seen that audio and tactile feedback facilitated performance in the similar manner as bimodal feedback. This suggests that the presentation of audio and tactile feedback alone was as effective in supporting task performance as bimodal feedback presentations.

The analysis of the subjective ratings of user’s perception in VR environments showed similarities to the objective measures of performance. Cue substitution in all modalities and the interactions between them had a significant effect on perceived sense of immersion and involvement, collectively known as presence [29–31]. Participants rated the multimodal cues as most effective in enhancing their sense of presence, followed by bimodal and then by unimodal substitute cues. The magnitude of these effects is significant and is confirmed by the effect sizes in our analysis, thus confirming that the availability of sensory cues during the VR interaction can influence users’ perception in VR environments [34, 40, 52]. When each mode of feedback is compared, the difference between bimodal and a unimodal feedback is minimal: this may be due to the fact that data were combined to form a unimodal category. The current study was not designed to show these particular differences, but one possible explanation for the difference between the modes of feedback may be due to the insufficient power in the experiment.

To investigate whether the objective performance was related to the users’ subjective experience, mean performance times and mean subjective ratings were used for the correlational analysis. The correlational analysis performed across all normalised data revealed a significant negative relationship between the objective performance measures (time to complete the task) and the users’ subjective experience. As previous studies have suggested [24, 33, 34, 42, 45, 52, 55, 62], these results are consistent with the claim that increased presence is correlated with higher performance in VR environments.

## Conclusion

In order to support training and performance in VR environments it is essential to provide necessary sensory cues that are required for the task. The results from our study show that substitute multimodal sensory feedback that might detract from the overall fidelity of VR environment can enhance overall task performance as well as the users’ perceived sense of presence. Tactile and auditory cues emerged as particularly useful substitute cues; they provided additional information in an efficient manner without being distractive, especially when participants needed to reach and grasp the objects during the VR interaction. We showed that tactile cues work well in combination with other cues (visual and audio), however as some differences between unimodal and bimodal signals were significant, a further investigation into the differential effects of these cues on user’s objective and subjective performance is encouraged. The results from correlational analyses showed that user’s subjective experience is related to the overall task performance: participants performed the task much faster and reported an increased sense of presence during the VR interaction. The correlation between the task performance and presence was expected as both of the factors have underlying cause of enhanced input. More detailed analysis, such as path analysis or partial correlation analysis, could determine the stability of this relationship; therefore future studies are encouraged to include these analyses to investigate whether the relationship between the factors is maintained. Additionally, our study showed that the use of subjective and objective measures for the evaluation of VR environments is worthwhile and is therefore recommended for future studies.

The substitute cues presented in this study were highly unrealistic; however they provided task-relevant information. The main implication stemming from this research implies that the addition of substitute sensory cues into the virtual environment can be beneficial even when the overall fidelity on the VR environment is decreased. Understanding of factors and conditions in multisensory cuing, under which VR users experience an enhanced sense of presence and increased performance, can help designers to allocate computational resources proportionally when building future designs of virtual systems with multimodal feedback [14, 33, 15, 18, 42–45, 48].

## Supporting information

S1 Dataset Raw data. (XLSX)

S1 File Objective data. (CSV)

S2 File Subjective data. (CSV)

S3 File Correlation. (PDF)

S4 File R script. (R)

S1 Video Multisensory substitute feedback in VR. (MP4)
