# Supplementary material for: The effects of substitute multisensory feedback on task performance and the sense of presence in a virtual reality environment
Source: PLoS One. 2018 Feb 1;13(2):e0191846. doi: 10.1371/journal.pone.0191846 (PMC5794113; doi:10.1371/journal.pone.0191846)
Supplement: S3 File — (PDF) [file pone.0191846.s004.pdf]

S1 File - Supplementary information

Correlation

Another way how to approach the statistical analysis on the data with a high variability in subjective measures is to average data across conditions. One of the aims of this study was to examine the effects of sensory condition on user subjective experience and their objective performance. As every participant completed every condition we assumed consistent dependency amongst them. Therefore for this correlation we pooled the data from each participant in every sensory condition to obtain a common mean for that condition. The correlation analysis was then performed between the objective and subjective data across sensory conditions and the result revealed a significant negative correlation ($r = -0.978$, $p < 0.001$) (Fig 12).

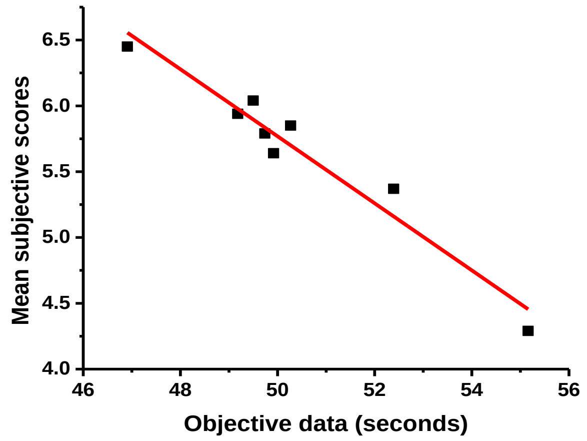

Fig 12. Correlation analysis across sensory conditions. The correlational analysis was performed on the normalised data across all sensory conditions ($r = -0.978$, $p < 0.001$).

Both of these approached show that higher scores on subjective ratings scale corresponded consistently with an improved task performance. It can be therefore concluded

that an enhanced sense of involvement and immersion, commonly also called presence can facilitate task performance in VR environment.
